# Sustaining and strengthening *biij*

**DOI:** 10.2349/biij.1.2.e8

**Published:** 2005-10-01

**Authors:** KH Ng, BJJ Abdullah

**Affiliations:** Department of Biomedical Imaging (Radiology), Faculty of Medicine, University of Malaya, Kuala Lumpur, Malaysia

As stated in the first issue, ***biij*** as an open access multidisciplinary online journal, “was set up to meet the challenges of biomedical imaging and intervention facing the allied sciences community by providing a new avenue for discussion and exchange of viewpoints.” The Biomedical Imaging and Intervention Journal (***biij***) was born on July 16th 2005 during the opening ceremony of the ‘Second International University of Malaya Research Imaging Symposium: Fundamentals of Molecular Imaging’. The multimedia launch was accompanied by Gustav Holst's Jupiter, the Bringer of Jollity. This is most fitting as applying advanced imaging and interventional techniques to improve the quality of health heralds joy to the world.

**Figure 1:**
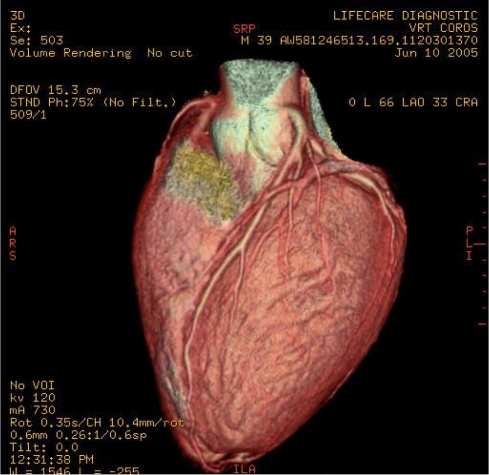
An example of a GIF animation showing a 3D volume rendering of the heart (courtesy of Dr. WF Liew).

**Figure 2:**
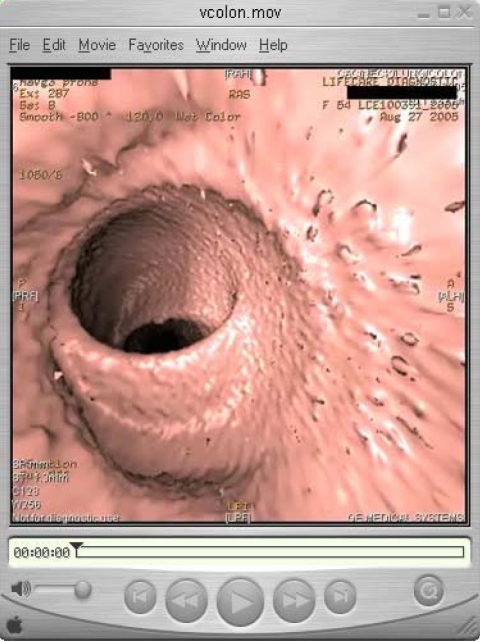
An example of a QuickTime movie showing a virtual colonoscopy (courtesy of Dr. WF Liew).

**Figure 3:**
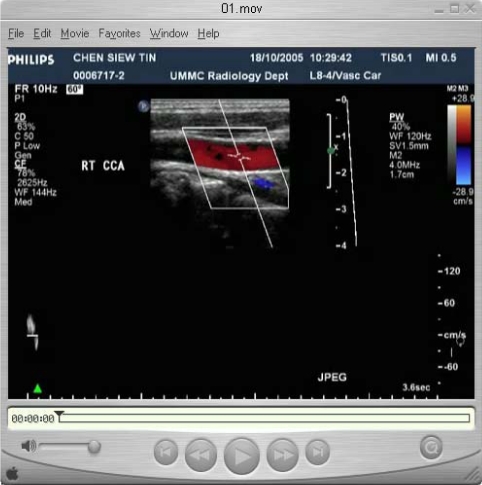
An example of a QuickTime movie showing a carotid Doppler ultrasound image (courtesy of Dr. A Vijayananthan).

We are especially interested in exploiting electronic publishing: online submission, online review, and online publication. Although numerous online journals have been published very few have taken advantage of the multimedia capabilities such as audio, video, animation, and simulation (Figures 1-3). We are convinced that this is the way journal publications will have to advance in the near future. Feature-rich html papers will appear in the ***biij***.

The abstracts of two international meetings have been archived in the first issue of the ***biij***, namely Asian Breast Diseases Association (ABDA) Third Teaching Course: Advances in the Management of Breast Diseases, Kuantan, Malaysia (28-30 May 2005) and the 2nd International University of Malaya Research Imaging Symposium: Fundamentals of Molecular Imaging, Kuala Lumpur, Malaysia (16-17 July 2005).

We are interested in archiving various abstracts and proceedings of scientific meetings. This serves as a depository of the various high-quality transactions.

Since its inception there have been a total of 3,136 page loads or visits to the journal web site; made by 640 first-time visitors and 269 returning visitors (as of 1 October 2005). We are pleased to have an international readership including Argentina, Australia, Austria, Canada, Chile, China, Egypt, Germany, Hong Kong, India, Indonesia, Japan, Jordan, Korea, Philippines, Singapore, Spain, Thailand, United Arab Emirates, United Kingdom, United States, Uruguay, and Vietnam.

Some of our readers have commented on its professional and polished appearance. From a modest start with four papers in the first issue we now have six papers in the second issue. Although this is an excellent start for a new journal we can do better.

We have already planned for special focus issues on radiotherapy, informatics, intervention and PET/CT. Well known researchers and medical scientists have been invited to write the guest editorials. We invite you to contribute.

The aspiration of ***biij*** is to attain the international standard and follow a stringent peer-review process. Our ultimate aim is to be indexed by various international bibliographic databases.

***biij*** has adopted the Uniform Requirements for Manuscripts Submitted to Biomedical Journals: Writing and Editing for Biomedical Publication (also known as the Vancouver Style) published by the International Committee of Medical Journal Editors [1]. Potential authors are encouraged to follow the instructions in accordance with the Uniform Requirements.

The success of a journal would have not been possible without the hard work of many people, including the Associate Editors, International Advisory Board and the Editorial Board, as well as all of the referees who take time to review the manuscripts. Finally we must not forget the corporations who sponsor the journal. Thanks to all of you.

Each of you also has a vital role to play in the further development of the journal by publicizing it among your friends and colleagues, submitting manuscripts for consideration to be published and supporting the sponsors.

We invite you to explore this innovative resource, and provide us with ideas and comments.
